# Photo Initiated Chemical Vapour Deposition To Increase Polymer Hydrophobicity

**DOI:** 10.1038/srep31574

**Published:** 2016-08-17

**Authors:** Ariane Bérard, Gregory S. Patience, Gérald Chouinard, Jason R. Tavares

**Affiliations:** 1 Department of Chemical Engineering, École Polytechnique de Montréal, Montreal, Quebec H3C 3A7, Canada; 2 Institut de Recherche et de Développement en Agroenvironnement, Saint-Bruno-de-Montarville, Quebec J3V 0G7, Canada

## Abstract

Apple growers face new challenges to produce organic apples and now many cover orchards with high-density polyethylene (HDPE) nets to exclude insects, rather than spraying insecticides. However, rainwater- associated wetness favours the development of apple scabs, *Venturia inaequalis*, whose lesions accumulate on the leaves and fruit causing unsightly spots. Treating the nets with a superhydrophobic coating should reduce the amount of water that passes through the net. Here we treat HDPE and polyethylene terephthalate using photo-initiated chemical vapour deposition (PICVD). We placed polymer samples in a quartz tube and passed a mixture of H_2_ and CO through it while a UVC lamp (254 nm) illuminated the surface. After the treatment, the contact angle between water droplets and the surface increased by an average of 20°. The contact angle of samples placed 70 cm from the entrance of the tube was higher than those at 45 cm and 20 cm. The PICVD-treated HDPE achieved a contact angle of 124°. Nets spray coated with a solvent-based commercial product achieved 180° but water ingress was, surprisingly, higher than that for nets with a lower contact angle.

Consumer demand for organic apples continues to increase. Leaves, branches and fruits are susceptible to pest infection and diseases. Several insecticide-free solutions have been proposed^1^,^2^,^3^. Sauphenor *et al.*^4^,^5^ cover trees with polymer nets to exclude insects, as was already practiced in Canada, France and Australia^5^,^6^,^7^. Despite their advantages, exclusion nets do not prevent important diseases from affecting apple trees. In particular, a pathogenic fungus, *Venturia inaequalis*, develops rapidly when fruit and foliage stay wet for prolonged periods. These infections, which also occur under nets because of their permeability, cause scab, a disease that generates dark spots on the fruit and reduces its commercial value^8^. Wind speed has an impact on drying time and propagation of infection^9^ and a large mesh size for exclusion nets is preferred to allow wind to pass through easily.

With a 160° contact angle and a low hysteresis of 10° 10, lotus leaves are the perfect example of pearling rain droplets. Micropapillae with branch-like nanostructures cover its surface and allows superhydrophobic behaviour^11^. To mimic lotus leaves, polymer exclusion nets should match these performance parameters. A high contact angle alone is insufficient for this application; the surface must also be non-adhesive. Droplets must trickle along the exclusion net (rather than pass through it), which is difficult when water adheres to the surface, as mentioned by Li *et al.*^12^.

Lee *et al.*^13^ presented static superhydrophobic behaviour for both water and oil droplets on 1 to 2 mm mesh size surfaces. They produced a polycyanoacrylate rectangular mesh with a 3D printer and studied the static behaviour – they placed droplets on the surface and measured the contact angle.

The Cassie-Baxter model explains this superhydrophobicity: the water droplets sit on air pockets, much like what happens on a lotus leaf’s microstructure^14^. A physical or chemical surface modification can provide this behaviour.

Advantages of physical treatment include basic processing and commercial accessibility^15^,^16^. To modify surfaces physically, several approaches are available such as electrospinning^17^, laser and chemical etching^18^,^19^,^20^,^21^ methods. Solvent treatments can also be used to physically alter a surface, by depositing new groups on the surface and forming superhydrophobic microstructures. Silicon-based commercial formulations, such as Rustoleum NeverWet, Ultra Ever Dry and Waterbeader, work according to this principle. However, in agricultural applications residual solvents or degradation products may leach out and contaminate the fruit, thus they are seldom used^17^,^22^.

Chemical modification is an alternative to this problem since the surface is functionalized. News groups are chemically bonded to the surface, which should prevent leaching. Available technologies included sol-gel^15^,^23^ and chemical vapour deposition (CVD)^24^. By opting for a gas-phase approach like CVD, it is possible to further prevent residual solvent leaching. Further, CVD coatings typically exhibit high film durability^25^ and are readily scaled up^26^,^27^. The deposition can be initiated by heat (thermally activated/TACVD)^28^,^29^, by plasma (plasma enhanced / PECVD)^30^,^31^ or by light (photo-initiated/PICVD)^32^,^33^. Heat initiation is problematic with temperature-sensitive substrates such as polymers^25^,^34^, whereas plasma is plagued with scale-up issues due to specific operating requirements^12^,^25^,^35^,^36^,^37^. PICVD is an alternative with its low energy treatment and wide spectrum of possible variations^24^,^35^. Indeed, depending on the light source used, it can be operated at ambient temperature and pressure conditions, without the use of highly specialized equipment. This article focuses on reducing the permeability of high-density polyethylene (HDPE) and polyethylene terephthalate (PET) nets via PICVD and evaluating their behaviour under simulated field conditions.

## Methods

### Experiments

#### Rain simulator

To demonstrate that a superhydrophobic net reduces water ingress during rainy days, we designed and built a rain simulator^38^ (see Supplementary Fig. S1). A peristaltic pump feeds water to a nozzle at a rate of 250 ml/h. Droplets with a diameter of 0.6 mm form at a needle placed at the end of the nozzle^39^. The droplets fall on a 1 mm by 0.6 mm exclusion net set at tilt angles ranging from 0° to 80°. The superhydrophobic coating (Rustoleum NeverWet) was applied following 2 steps. First, a base coat was sprayed approximately 15 cm to 30 cm from the net. After 30 min, a topcoat was sprayed at the same distance. After an additional 30 min the test was initiated.

#### Photo initiated chemical vapour deposition (PICVD)

Polymer samples are placed at various lengths along a 25 mm ID quartz tube (see Supplementary Fig. S2). Argon (400 ml min^−1^) purged the tube for 2 min to scavenge residual oxygen. Brooks mass flow controllers metered the gases to the reactor (400 ml min^−1^). Two 30 W UVC lamps (253.7 nm, irradiance of 5.5 × 10^−4^ μW cm^−2^, at 4.5 cm) illuminated the surface of the polymer samples. A precision valve controlled the pressure in the tube. The line was connected to a three-way valve leading to vacuum to test sub atmospheric pressures. For further details, see Dion *et al.*^40^. The factors tested to functionalize the polymer surfaces included time, pressure, H_2_/CO (syngas) ratio, photo initiator feed rate (H_2_O_2_) as well as sample position in the reactor (3 to 87 cm from the inlet). Ranges for each factor can be found in Supplementary Table S1. We tested both HDPE, which apple growers use to cover their trees, as well as PET. HDPE exclusion nets typically contain a UV protector (used to extend the life-span of the net itself), but that protector limits the reaction rate initiated by the UVC lamp. Therefore, PICVD assays were conducted with PET, which has superior UV resistance, and HDPE sheets without the UV protector package.

## Materials

Samples included: Protek Net 80 g/m^2^ HDPE exclusion net (Dubois Agrinovation) with a 1 mm by 0.6 mm mesh size, which is optimal for apple tree applications; sheet extrusion HDPE (McMaster) for PICVD experiments cut, to 15 mm by 38 mm by 1.3 mm; and film PET (McMaster) for PICVD tests, cut to 15 mm by 38 mm by 0.3 mm. CO, H_2_ and argon were purchased from Air Liquide and hydrogen peroxide (50%) from Sigma-Aldrich.

All polymer samples were washed in a Fisher Scientific ultrasonic bath (model FS110H) in two steps. First, samples were submerged in water for 15 min, followed by an acetone bath. Samples were then dried in a vacuum desiccator for at least 2 h.

### Characterization

#### Contact angle measurement

After each experiment, 5 contact angle measurements were taken per sample and the average value is reported. 2 μL of distilled water is deposited on the untreated or treated surface to measure the sessile drop contact angle. FDS tensiometer OCA DataPhysics TBU 90E recorded all contact angles after one minute from when the drop reaches the surface.

#### Fourier Transform Infrared Spectroscopy (FTIR)

A Thermo Scientific Nicolet iS5 Spectrometer equipped with an ATR module measured the infrared absorbance as a function of wave number. The analysis range varied from 400 cm^−1^ to 4000 cm^−1^ and the number of scans was set to 16 with a resolution of 4 cm^−1^.

#### Atomic Force Microscopy (AFM)

Atomic force microscopy images were collected with a multiMode8 AFM, Bruker/Santa Barbara NanoScope V, using standard tapping mode with a Tespa-type needle, in air at room temperature. Intermittent contact imaging was performed at a scan rate of 1 Hz using etched silicon cantilevers (ACTA from AppNano) with a resonance frequency around 300 kHz (spring constant of ≈42 N/m and tip radius of <10 nm). Images were taken with a 512 × 512 pixel resolution over a 5 μm × 5 μm square with medium tip oscillation damping (20–30%).

## Results and Discussion

### Hypothesis validation

**Figure 1:**
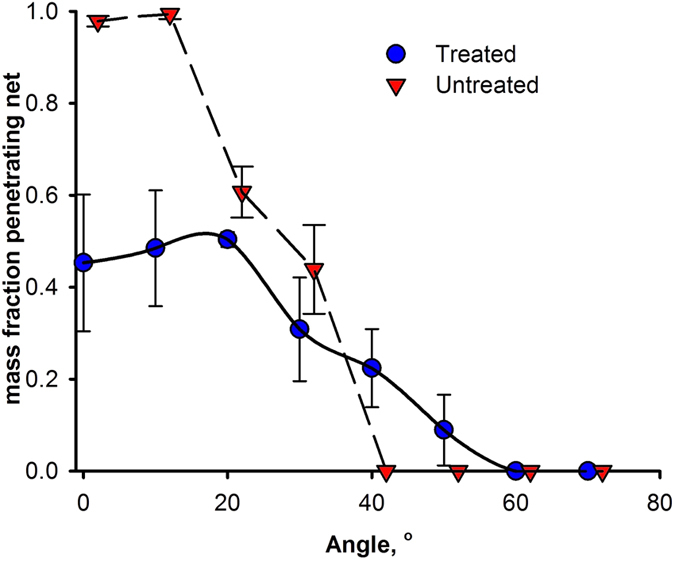
Water fraction passing through an HDPE exclusion net as a function of tilt angle. Error bars represent the standard deviation (*n* = 5).

**Figure 2:**
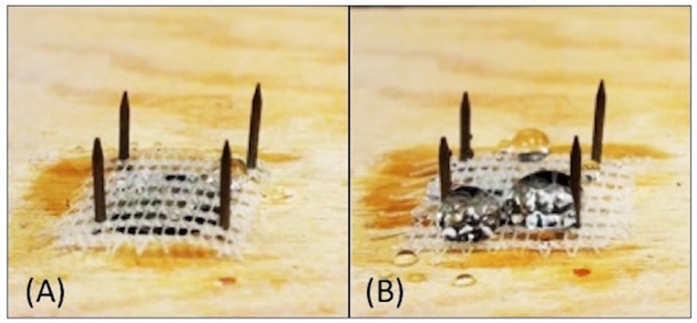
Rain test on HDPE exclusion net at a tilt angle of 0°. (**A**) Untreated HDPE, (**B**) Treated with silicon superhydrophobic coating.

Despite the Lee *et al.* work, it is not intuitive that a superhydrophobic surface can prevent water ingress through a net. To prove this concept, a rain simulator was built to simulate rain fall on real HDPE exclusion nets. Figures 1 and 2 show how a superhydrophobic net treated with a silicon-based coating can limit water ingress. The commercial treatment reduces the amount of water passing through the net by 70%, even in a horizontal position. The major problem with this solution is treatment longevity. After about 2 h the commercial coating disappears. Moreover, the treated HDPE net loses its mechanical properties such as elasticity– the net becomes visibly stiffer (Supplementary Video S1). This would be problematic when it becomes windy. Given that PICVD coatings are thinner than silicon-based treatments, the degradation rates should be reduced considerably.

### Experimental Design

An experimental design was conducted and related to statistical models to gain a better understanding of the effect of operating conditions as a function of the substrate selected. These models were employed to target the optimal conditions for the highest contact angle. The contact angle results following an experimental design using multiple approaches are presented in Supplementary Table S2. Initially, the contact angle on HDPE without a treatment is 96° ± 3° and for PET it is 85° ± 3°. A combination of 3 experimental plans was developed to account for quadratic effects. We designed a fractional factorial design (FFD) of resolution V to account for linear affects. Resolution V allows having fewer experiments with greater precision^41^. The central composite design (CCD) is complementary to the FFD, as it takes into account quadratic effects^42^. The Box-Benhken plan added experiments to identify additional quadratic effects^43^. Finally, midpoint experiments fixed the model in space^44^. Contact angle results presented are the average from 5 measurements per sample. When a value is aberrant according to Chauvenet’s criterion, it is deleted^45^.

### Model Equation

**Figure 3:**
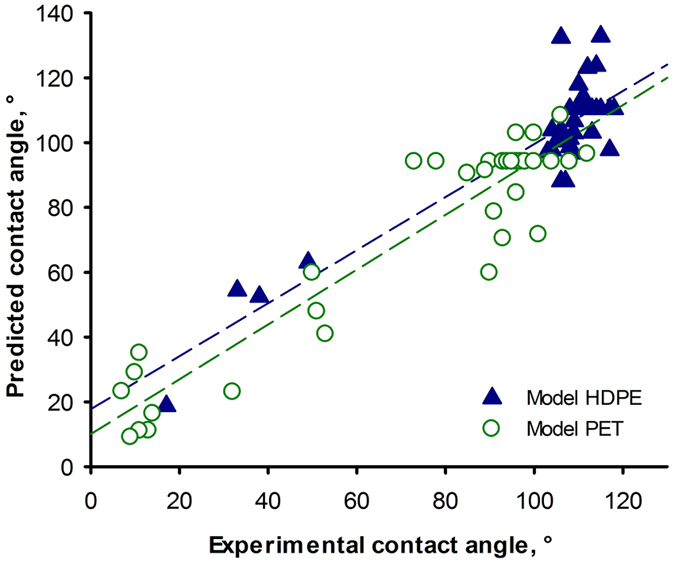


Dion *et al.*^40^ presented a statistical model for the PICVD treatment of copper with a similar experimental design. The major contributing factors to the model were position and syngas ratio. We tested five factors– pressure, time, position, H_2_/CO ratio and H_2_O_2_ and developed a regression model based on the Marquardt-Levenberg algorithm to identify a model that maximizes the multiple R^2^ (*SigmaPlot*^®^). Interactions between each variable and the possibility of second-order effects were verified. Only the significant variables and interactions were kept in the model (equation (1) for HDPE and equation (2) for PET). Parameter values can be found as Supplementary Tables S3 and S4. In each model, an aberrant point was deleted according to a large deviation from the quartile^46^ and we normalized the factors (supplementary information). The fact that a value has been removed from the model leads to the conclusion that these models are an approximation of the behaviour after the surface modification. Despite the high value of the regression coefficients (R^2^), the models are accurate only inside the range studied (Fig. 3).


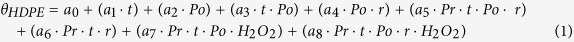



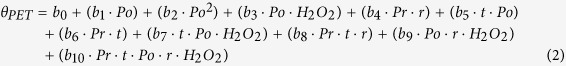


### Comparison of the two models

The two models seem very complex, evidenced by a large number of different factors that influence the reaction. Moreover, the parameters are different, indicating that the reaction depends on the substrate. The majority of experiments for HDPE led to hydrophobic behaviour, explaining the mass of data points between 100 and 120. For PET, the variance is slightly higher and the results are distributed over all contact angles values.

Position (Po) appears to be a key factor in the surface modification process, having an individual effect on the reaction, as well as many interactions with others factors. Most of these interactions are linear, except for one quadratic effect in the PET model. Pressure (Pr), H_2_/CO ratio (r) and hydrogen peroxide (H_2_O_2_) must be combined with other factors to influence the reaction. In both models, the interaction between the five factors cannot be omitted.

### First approach to kinetic modelling

**Figure 4:**
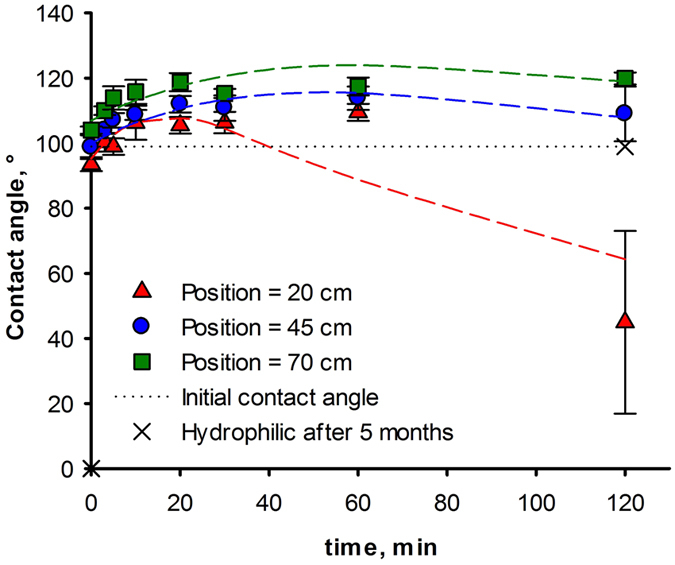


Position is the most significant factor in the PICVD reaction and must be analyzed in depth. To more clearly illustrate its interaction with treatment time (and infer reaction kinetics), samples were placed in the reactor for 3 min, the contact angle measurement was recorded and the samples were returned to the reactor for treatment for an additional time increment and so on. Figure 4 present the contact angle as a function of time for HDPE. Supplementary Fig. S3 shows similar figure for PET. For each experiment, the ratio H_2_/CO was 0.12 with 1 ml/h peroxide injected at 10 kPa relative pressure.

We can express the evolution in contact angle as a function of time as a sum of two reactions, the first one decreasing the contact angle (k_1_) and the second one increasing it (k_2_). Assuming first order reactions, we obtain:


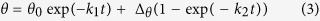


where θ_0_ is the initial contact angle and Δ_θ_ is the coefficient of variation of the contact angle. The fit parameters are tabulated in Table 1.

Despite somewhat low R^2^ values (attributed to data variation), this preliminary kinetic modelling highlights the two different reactions that occur in PICVD. First, k_2_ is always higher than k_1_ which indicates that the reaction leading to a contact angle increase is the fastest. On the other hand, the decreasing reaction’s pre-exponential factor, θ_0_ is higher, implying that it will become dominant over time. As position in the reactor increases, the coefficients k_1_ and k_2_ stabilize – in other words, the difference in contact angle will be minimal if the samples are further inserted from the gas inlet. For the two polymers, the k_1_ and k_2_ coefficients are similar, hence the same measured increase in contact angle (approximately 20°). The coefficient of variation of the contact angle (Δθ_0_) is identical for all substrates and positions, and represents the maximum variation attainable. Moreover, there are diminishing returns on treatment: the contact angle will not change significantly after a certain treatment time. A significant decrease in contact angle is observable at position Po = 20 cm for each polymer after 1 hour. The high variability between the model presented and these data points can be explained by hydrophobic recovery. Indeed, surfaces treated to become hydrophilic tend to return to their native contact angle values within hours or days^47^. Therefore, the hydrophilic contact angle values reported do not represent their steady state values. After 5 months, hydrophilic-treated surfaces return to the native contact angle of the substrate, while hydrophobic-treated surfaces retain their behaviour (constant contact angle). A model based on the concentration of the reagents as a function of time and reactor position be needed to gain greater understanding of the reaction kinetics.

### Extreme values

**Figure 5:**
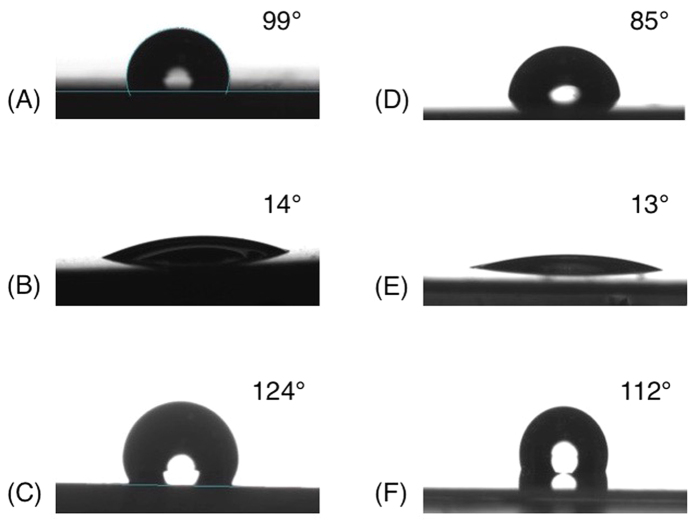
Extreme contact angle values. (**A**) Untreated HDPE (**B**) Hydrophilic treated HDPE: Pr = −10 kPa relative pressure, t = 60 min, r = ½, Po = 20 cm and H_2_O_2_ = 1 mL/h (**C**) Hydrophobic treated HDPE: Pr = 10 kPa relative pressure, t = 30 min, r = ½, Po = 20 cm and H_2_O_2_ = 0 mL/h (**D**) Untreated PET (**E**) Hydrophilic treated PET: Pr = 0 kPa relative pressure, t = 75 min, r = 2.06, Po = 20 cm and H_2_O_2_ = 1 mL/h. (**F**) Hydrophobic treated PET: Pr = 10 kPa relative pressure, t = 120 min, r = 0.12 and Po = 70 cm.

The experimental plan allowed for a mapping of the various surface properties that can be obtained on HDPE and PET following PICVD treatment. Table 2 and Fig. 5 highlight the extreme values (i.e. most hydrophobic and most hydrophilic behaviour) that could be obtained by PICVD.

Many experimental conditions can yield hydrophilic surfaces on both polymers, but this effect is unstable because of hydrophobic recovery^47^. Moreover, while the typical target for superhydrophobicity is a contact angle higher than 150°, the more moderate increase in hydrophobicity achievable by PICVD does affect water ingress (see section “exclusion net experiments”).

### Fourier Transform Infrared Spectroscopy (FTIR)

**Figure 6:**
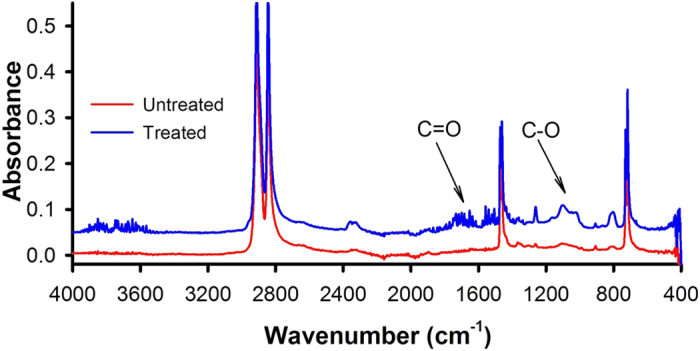
FTIR absorbance for untreated and hydrophobic treated HDPE. Experimental conditions for treated sample: pressure = 10 kPa relative pressure, ratio H_2_/CO = 0.5, time = 30 min, position = reactor inlet with no hydrogen peroxide.

HDPE is a linear molecule with only C-H groups. After hydrophobic PICVD treatment, new functional groups appear (see Fig. 6). Indeed, C-O links are present after treatment, evidenced by new peaks around 1100 cm^−1^, which can be attributed to alcohol, or ether groups. Moreover, C=O bonds are identified around 1600 cm^−1^, in the form of ketone or aldehydes. The combination of peaks ca. 1100 cm^−1^ and 1600 cm^−1^ are a strong indication of carboxylic functionality^48^,^49^.

Despite the measured contact angle difference, no visible differences can be measured by FTIR for PET, likely due to the presence of oxygen atoms in its molecular structure before any treatment. A more surface-sensitive analysis technique such as X-ray Photoelectron Spectroscopy (XPS) would be needed to probe the functional groups imparted by PICVD.

### Atomic Force Microscope (AFM)

**Figure 7:**
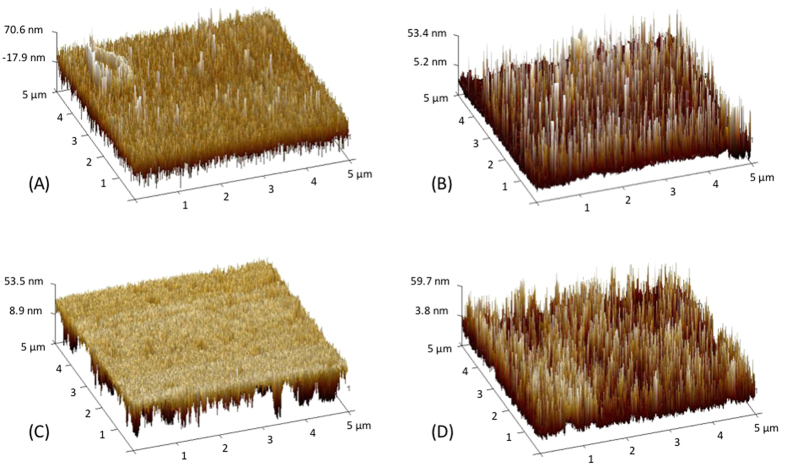
AFM Images (**A**) Untreated HDPE 3D (5 μm × 5 μm) (**B**) Treated HDPE 3D (5 μm × 5 μm) Experimental conditions for treated sample: pressure = 10 kPa relative pressure, ratio H2/CO = 0.5, time = 30 min., position = 20 cm with no hydrogen peroxide (**C**) Untreated PET 3D (5 μm × 5 μm) (**D**) Treated PET 3D (5 μm × 5 μm). Experimental conditions for treated sample: pressure = 10 kPa relative pressure, ratio H_2_/CO = 0.12, time = 120 min, position = 70 cm with no hydrogen peroxide.

It is somewhat counter-intuitive that contact angles increase with the addition of oxygen-containing functional groups. However, roughness plays a significant role in hydrophobicity. This can be measured through AFM (see Fig. 7). While both polymers are initially quite flat, PICVD treatment leads to the appearance of several “islands”. This radical change in roughness increases the formation of air pockets below the water droplets, thus leading to the observed hydrophobic behaviour^50^,^51^. For PET, the AFM images provide conclusive evidence of surface modification, despite the absence of change in the FTIR spectra. PET roughness increased after PICVD treatment, while this change was more moderate for HDPE. Quantitative characterisation for both polymers is presented in supplementary Table S5.

### Exclusion net experiments

**Figure 8:**
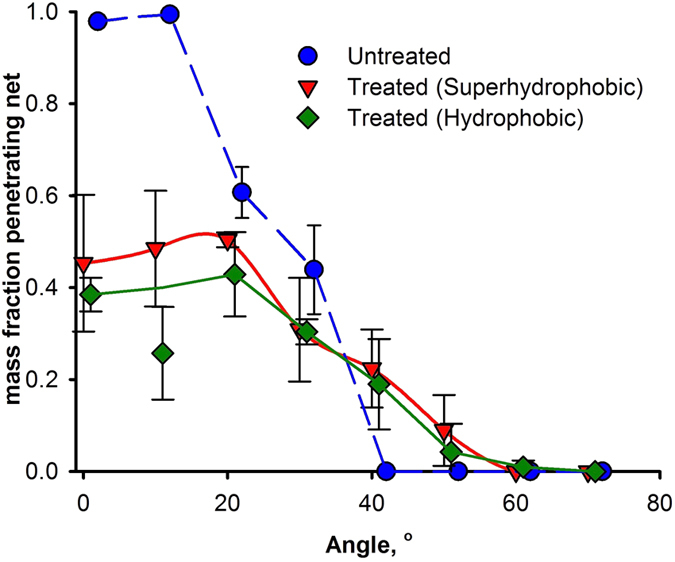
Water fraction passing through an HDPE exclusion net (mesh size 1.0 × 0.60 mm) as a function of tilt angle without treatment and with a simulated hydrophobic treatment. Error bars represent the standard deviation.

The contact angle reached a maximum of 124° by PICVD on HDPE (without any UV protection package). Because the HDPE exclusion nets contain a UV protector, they could not be treated in the PICVD reactor. However, applying the commercial single step “Rustoleum NeverWet for Outdoor Fabric Treatment” formulation could simulate this hydrophobic effect. We used this product instead of the original NeverWet product because the contact angle on the exclusion nets reached 120°. After applying the coating in a single step and drying for 24 h, rain tests were conducted (see Supplementary Fig. S1). The results for this “less” hydrophobic treatment are nearly identical to that of the superhydrophobic treatment (see Fig. 8). Although the HDPE nets could not be directly treated by PICVD, this simulated behaviour demonstrates that, if the UV protector issue can be curtailed, a 120° contact angle should be sufficient to prevent water ingress on exclusion nets, at least under conditions similar to those of our rain simulator.

The results for superhydrophobic and hydrophobic treatment are statistically indistinguishable above 20°: the error bars overlap and the average water fraction that penetrates is within 5%. At 10°, the superphydrophobic surface allows more water to pass through the net (p < 0.05). On the superhydrophobic treated nets, the droplets bounce many times on the surface. This may increase the chance that smaller droplets form during rebound and these pass through the holes. So, a hydrophobic surface with a contact angle of 120° is good enough to minimize water passing through the studied exclusion nets.

## Conclusions

High contact angle exclusion nets for organic apple application are an alternative to minimize fungicides that are otherwise needed to control apple scabs. The PICVD reaction renders polymer surfaces hydrophobic, which is desirable to minimize rainwater ingress to the trees, but also hydrophilic depending on the treatment conditions. To optimize the hydrophobicity, we derived a mathematical relationship for each polymer based on an experimental design. We measured the contact angle as a function of exposure time and fit the data to a differential equation. FTIR and AFM confirmed that the surface was chemically modified. Rain test results showed that a superhydrophobic or a hydrophobic coating minimized water ingress through a ProtekNet 80g (mesh size 1.0 × 0.60 mm). A more precise study on surface degradation with time, sun, humidity and wind is required to establish the suitability of this technology for commercialization.

## Additional Information

**How to cite this article**: Bérard, A. *et al.* Photo Initiated Chemical Vapour Deposition To Increase Polymer Hydrophobicity. *Sci. Rep.* **6**, 31574; doi: 10.1038/srep31574 (2016).

## Supplementary Material

Supplementary Information

Supplementary Video S1

## Figures and Tables

**Table 1:** Parameters for kinetic modelling (equation (3)).

	Position [cm]	θ_0_ [°]	Δθ_0_[°]	k_1_ [min^−1^]	k_2_ [min^−1^]	R^2^ (%)
HDPE	20	100	41.6	0.011	0.071	74
	45	100	41.6	0.003	0.026	79
	70	100	41.6	0.003	0.024	60
PET	20	85	41.6	0.011	0.071	60
	45	85	41.6	0.002	0.041	72
	70	85	41.6	0.002	0.024	80

**Table 2:** Highest and lowest contact angle values obtained for PET and HDPE.

	HDPE		PET	
	Contact angle [θ_HDPE_] (°)	Standard deviation (°)	Contact angle [θ_PET_] (°)	Standard deviation (°)
Hydrophobic treated	124	±3	112	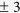
Hydrophilic treated	14	±2	13	±1
