# Corrigendum: Photo Initiated Chemical Vapour Deposition To Increase Polymer Hydrophobicity

**DOI:** 10.1038/srep39422

**Published:** 2017-02-17

**Authors:** Ariane Bérard, Gregory S. Patience, Gérald Chouinard, Jason R. Tavares

Scientific Reports 6: Article number: 3157410.1038/srep31574; published online: 08 17 2016; updated: 02 17 2017

This Article contains an error in the Methods section. “Two 30 W UVC lamps (253.7 nm, irradiance of 5.5 × 10^–4^ μ W cm^−2^, at 4.5 cm) illuminated the surface of the polymer samples”.

should read:

“Two 30 W UVC lamps (253.7 nm, irradiance of 0.01 W cm^−2^, at 3.5 cm) illuminated the surface of the polymer samples”.

