# Supplementary material for: Photo Initiated Chemical Vapour Deposition To Increase Polymer Hydrophobicity
Source: Sci Rep. 2016 Aug 17;6:31574. doi: 10.1038/srep31574 (PMC4987658; doi:10.1038/srep31574)
Supplement: Supplementary Information [file srep31574-s1.doc]

**Photo Initiated Chemical Vapour Deposition To Increase Polymer Hydrophobicity**

Ariane Bérard, Gérald Chouinard, Gregory S. Patience, Jason R. Tavares*

Supplementary information


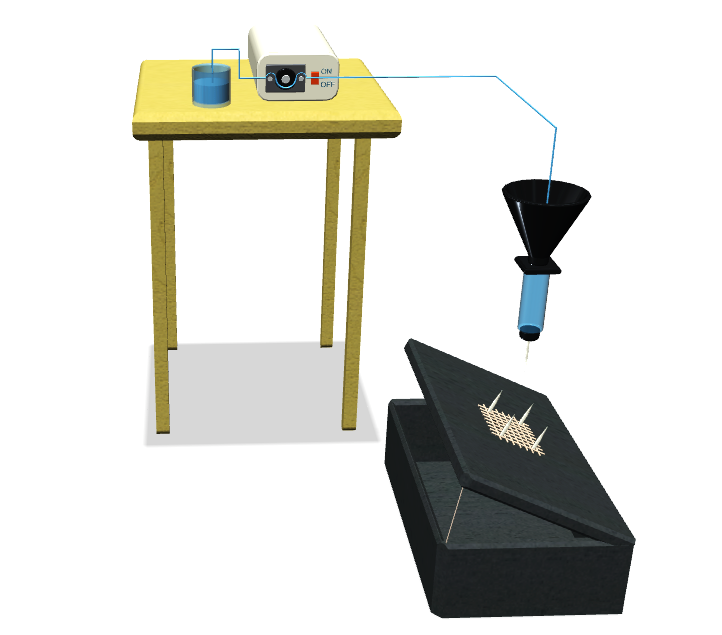


Supplementary Figure S1: Rain simulator set up (Autodesk 123D®. Autodesk, AutoCAD, DWG, the DWG logo, and Inventor are registered trademarks or trademarks of Autodesk, Inc., and/or its subsidiaries and/or affiliates in the USA and other countries.)

Supplementary Table S1: PICVD parameter ranges

|  | Range |
| --- | --- |
| Pressure | -17 to 17 kPa |
| Sample position | 3 to 87 cm from gas inlet |
| Ratio H_2_/CO | 0 to 6.7 |
| Time | 0 to 151 minutes |
| Hydrogen peroxide | 0 to 1 ml/h |

Supplementary Figure S2: PICVD process diagram

Supplementary Table S2: Contact angle results for HDPE and PET following the three experimental designs.

|  | No Exp | Time  (min)  [t] | Pressure  (kPa)  [Pr] | Ratio H_2_/CO  [r] | Position  (cm)  [Po] | H_2_O_2_  (ml/h)  [H_2_O_2_] | Contact angle HDPE (°) [θ_HDPE_] | Contact angle PET (°)  [θ_PET_] |
| --- | --- | --- | --- | --- | --- | --- | --- | --- |
| Fractional  Factorial  Design  Resolution  V | 1 | 120 | + 10 | 4 | 70 | 1 | 106 ± 3 | 96 ± 1 |
|  | 2 | 30 | + 10 | 4 | 20 | 1 | 107 ± 3 | 101 ± 3 |
|  | 3 | 120 | - 10 | 4 | 20 | 1 | 33 ± 9 | 11 ± 1 |
|  | 4 | 30 | - 10 | 4 | 70 | 1 | 105 ± 1 | 51 ± 6 |
|  | 5 | 120 | + 10 | 0.12 | 20 | 1 | 17 ± 8 | 7 ± 2 |
|  | 6 | 30 | + 10 | 0.12 | 70 | 1 | 113 ± 8 | 90 ± 7 |
|  | 7 | 120 | - 10 | 0.12 | 70 | 1 | 109 ± 2 | 50 ± 10 |
|  | 8 | 30 | - 10 | 0.12 | 20 | 1 | 40 ± 10 | 50 ± 10 |
|  | 9 | 120 | + 10 | 4 | 20 | 0 | 108 ± 8 | 13 ± 3 |
|  | 10 | 30 | + 10 | 4 | 70 | 0 | 104 ± 4 | 85 ± 6 |
|  | 11 | 120 | - 10 | 4 | 70 | 0 | 108 ± 2 | 106 ± 4 |
|  | 12 | 30 | - 10 | 4 | 20 | 0 | 106 ± 3 | 10 ± 2 |
|  | 13 | 120 | + 10 | 0.12 | 70 | 0 | 104 ± 7 | 112 ± 3 |
|  | 14 | 30 | + 10 | 0.12 | 20 | 0 | 109 ± 9 | 53 ± 3 |
|  | 15 | 120 | - 10 | 0.12 | 20 | 0 | 49 ± 3 | 30 ± 10 |
|  | 16 | 30 | - 10 | 0.12 | 70 | 0 | 106 ± 5 | 91 ± 3 |
| Central Composite  Design | 17 | 151 | 0 | 2.06 | 45 | 0 | 117 ± 5 | 78 ± 2 |
|  | 18 | 0 | 0 | 2.06 | 45 | 0 | 96 ± 2 | 84 ± 3 |
|  | 19 | 75 | 17 | 2.06 | 45 | 0 | 115 ± 2 | 97 ± 5 |
|  | 20 | 75 | - 17 | 2.06 | 45 | 0 | 111 ± 5 | 78 ± 4 |
|  | 21 | 75 | 0 | 6.7 | 45 | 1 | 108 ± 2 | 73 ± 6 |
|  | 22 | 75 | 0 | 0 | 45 | 1 | 96 ± 4 | 87 ± 1 |
|  | 23 | 75 | 0 | 2.06 | 87 | 1 | 115 ±5 | 14 ± 2 |
|  | 24 | 75 | 0 | 2.06 | 3 | 1 | 106 ± 2 | 89 ± 5 |
| Box-Benhken | 25 | 75 | 0 | 2.06 | 20 | 1 | 103 ± 5 | 13 ± 1 |
|  | 26 | 75 | 0 | 4 | 70 | 0.5 | 111 ± 4 | 96 ± 6 |
|  | 27 | 75 | 0 | 0.12 | 45 | 0 | 113 ± 3 | 96 ± 3 |
|  | 28 | 75 | + 10 | 2.06 | 70 | 0.5 | 114 ± 3 | 100 ± 9 |
|  | 29 | 75 | + 10 | 2.06 | 45 | 0 | 115 ± 8 | 100 ± 1 |
|  | 30 | 75 | - 10 | 4 | 45 | 0.5 | 111 ± 5 | 11 ± 2 |
|  | 31 | 30 | 0 | 2.06 | 20 | 0.5 | 111 ± 3 | 93 ± 8 |
|  | 32 | 30 | 0 | 2.06 | 45 | 1 | 110 ± 2 | 95 ± 4 |
|  | 33 | 120 | 0 | 0.12 | 45 | 0.5 | 109 ± 1 | 108 ± 7 |
|  | 34 | 120 | - 10 | 2.06 | 45 | 0.5 | 69 ± 5 | 9 ± 1 |
| Midpoint | 35 | 75 | 0 | 2.06 | 45 | 0.5 | 110 ± 6 | 97 ± 5 |
|  | 36 | 75 | 0 | 2.06 | 45 | 0 | 112 ± 4 | 93 ± 7 |
|  | 37 | 75 | 0 | 2.06 | 45 | 1 | 117 ± 3 | 98 ± 4 |
|  | 38 | 75 | 0 | 2.06 | 45 | 0.5 | 118 ± 5 | 94 ± 4 |
|  | 39 | 75 | 0 | 2.06 | 45 | 0 | 113 ± 4 | 95 ± 6 |
|  | 40 | 75 | 0 | 2.06 | 45 | 1 | 114 ± 3 | 100 ± 4 |

Legend: Contact angle uncertainties provide from standard deviation.

Supplementary Table S3: Parameters values for HDPE model

| Parameters a_i_ | | | | | |
| --- | --- | --- | --- | --- | --- |
| a_0_ = | 110 | a_3_ = | 9.3 | a_6_ = | 12 |
| a_1_ = | -7.5 | a_4_ = | -9.6 | a_7_ = | 8.3 |
| a_2_ = | 13 | a_5_ = | -10 | a_8_ = | -22 |

Supplementary Table S4: Parameters values for PET model

| Parameters b_i_ | | | | | |
| --- | --- | --- | --- | --- | --- |
| b_0_ = | 94 | b_4_ = | 83 | b_8_ = | 35 |
| b_1_ = | 23 | b_5_ = | 14 | b_9_ = | -91 |
| b_2_ = | -14 | b_6_ = | 85 | b_10_ = | -20 |
| b_3_ = | -45 | b_7_ = | -78 |  |  |

Supplementary equations: normalization

$t=\frac{time-75}{45}$

$Pr=\frac{pressure}{10}$ (2)

$r=\frac{ratio-2.06}{1.94}$

$H_{2}O_{2}=\frac{peroxide-0.5}{0.5}$

$Po=\frac{position-45}{25}$

Supplementary Figure S3: Contact angle as a function of time for three different positions (PET model)

Supplementary Table S5: Quantitative results for AFM

|  | Untreated HDPE | Treated HDPE | Untreated PET | Treated PET |
| --- | --- | --- | --- | --- |
| Surface area (μm^2^) | 26.3 | 25.3 | 25.5 | 25.9 |
| Projected surface area (μm^2^) | 25.0 | 25.0 | 25.0 | 25.0 |
| Surface area difference (%) | 5.03 | 1.07 | 2.17 | 3.16 |
| R_q_ (nm) | 17.7 | 17.4 | 10.8 | 17.8 |
| R_a_ (nm) | 10.9 | 13.0 | 7.10 | 14.8 |

Supplementary Video S1: Rain simulator proof of concept
